# O001: Getting the unexpected: no association between hand hygiene and workload

**DOI:** 10.1186/2047-2994-2-S1-O1

**Published:** 2013-06-20

**Authors:** S Scheithauer, M Dangel, B Batzer, C Pino Molina, A Widmer

**Affiliations:** 1 Infection Control, University Hospital Basel, Basel, Switzerland; 2 Infection Control & Infectious Diseases, University Hospital Aachen, RWTH Aachen, Aachen, Germany; 3 Hematology, University Hospital Basel, Basel, Switzerland

## Introduction

A high compliance with hand hygiene is a cornerstone of any infection control program. However, a high workload and a lack of time are most commonly used argues against an appropriate compliance.

## Objectives

In order to assess the relationship between the hand hygiene events (HHE) and the workload, we correlated HHEs per patient-day (PD) with the staff time/PD (h), the nursing effort/PD (h) and the C-value indexing the workload, respectively.

## Methods

All HHEs at a hematology ward (University Hospital Basel, Switzerland) were continuously recorded from 01.03.12 to 28.02.213 using the Ingo-man Weco (Ophardt Hygienetechnik, Issum; Germany) and could be analyzed dispenser-, day-, shift-, localization-specifically. Daily data on patients, staff time (h), nursing effort (h), C-value (1 – (nursing effort / weighted staff time)*100) were calculated with regard to the workday from the electronic patient documentation sheets. For statistics SPSS was used.

## Results

During the one year investigation 208.184 HHE translating into 57 (±10) HHE/PD were performed. HHE from Monday to Friday exceeded HHE during the weekends with 59 (±10) versus 51 (±9) /PD. HHE/PD were significantly associated with the staff time with r=0.37 (p=0.01) and with the nursing effort with r=0.41 (p=0.01), respectively. These associations could be verified during workdays as well as during the weekends. In contrary, HHE/PD did not depend on workload in general indexed by the C-value with r=-0.04. However, during Monday and Friday HHE/PD seemed to correlate even inversely with the C-value (r=0.20; p=0.01).

## Conclusion

HHE/PD were associated with the staff time and the nursing effort indicating a constant compliance regardless the workload. This hypothesis was confirmed by the lack of a positive association between the C-value and the HHE/PD. Thus compliance seemed not to be affected by workload at the hematology ward enrolled in this investigation.

## Disclosure of interest

None declared.

